# Thalamo-cortical networks in subtypes of migraine with aura patients

**DOI:** 10.1186/s10194-021-01272-0

**Published:** 2021-06-19

**Authors:** Gianluca Coppola, Antonio Di Renzo, Emanuele Tinelli, Barbara Petolicchio, Vincenzo Parisi, Mariano Serrao, Camillo Porcaro, Marco Fiorelli, Francesca Caramia, Jean Schoenen, Vittorio Di Piero, Francesco Pierelli

**Affiliations:** 1 Department of Medico-Surgical Sciences and Biotechnologies, Sapienza University of Rome Polo Pontino, Corso della Repubblica 79, 04100 Latina, Italy; 2 IRCCS – Fondazione Bietti, Rome, Italy; 3 Department of Human Neurosciences, Sapienza University of Rome, Rome, Italy; 4 Institute of Cognitive Sciences and Technologies (ISTC) - National Research Council (CNR), Rome, Italy; 5 S. Anna Institute and Research in Advanced Neurorehabilitation (RAN), Crotone, Italy; 6 Headache Research Unit, University Department of Neurology CHR, Citadelle Hospital, University of Liège, Liège, Belgium; 7 IRCCS - Neuromed, Pozzilli, IS Italy

**Keywords:** migraine, aura, resting state, diffusion tensor imaging, thalamus, networks

## Abstract

**Background:**

We searched for differences in resting-state functional connectivity (FC) between brain networks and its relationship with the microstructure of the thalamus between migraine with pure visual auras (MA), and migraine with complex neurological auras (MA+), i.e. with the addition of at least one of sensory or language symptom.

**Methods:**

3T MRI data were obtained from 20 patients with MA and 15 with MA + and compared with those from 19 healthy controls (HCs). We collected resting state data among independent component networks. Diffusivity metrics of bilateral thalami were calculated and correlated with resting state ICs-Z-scores.

**Results:**

As compared to HCs, both patients with MA and MA + disclosed disrupted FC between the default mode network (DMN) and the right dorsal attention system (DAS). The MA + subgroup had lower microstructural metrics than both HCs and the MA subgroup, which correlated negatively with the strength of DMN connectivity. Although the microstructural metrics of MA patients did not differ from those of HCs, these patients lacked the correlation with the strength of DAS connectivity found in HCs.

**Conclusions:**

The present findings suggest that, as far as MRI profiles are concerned, the two clinical phenotypes of migraine with aura have both common and distinct morpho-functional features of nodes in the thalamo-cortical network.

## Introduction

Approximately 30 % of migraine patients have an aura that precedes or accompanies the headache phase [1]. Migraine auras consist of visual symptoms in up to 98 % of cases, with the addition of sensory symptoms in 36 %, and language dysfunction in 10 % of cases [2]. It was suggested that migraine with aura might be a heterogeneous condition where different pathophysiological mechanisms could explain the variable clinical phenotype [3]. Supporting this hypothesis, distinct neurophysiological [4, 5] and MRI [6, 7] abnormalities of the visual cortex were detected in patients with complex neurological auras where visual symptoms are associated with sensory and/or dysphasic symptoms. In migraine with aura between attacks, without distinction of aura subtypes, previous studies obtained evidence for aberrant thalamic and thalamocortical fiber microstructure [8–12], as well as for abnormal cortical functional connectivity [12–16]. Hence, whether specific thalamo-cortical network abnormalities may exist in patients with complex auras, compared those with purely visual auras, or healthy controls, is a research question of great interest. Clarifying it may indeed help understanding the mechanisms underlying the different clinical expression of migraine auras.

It is known that brain disorders can affect one or more structural (micro- or macroscopic) and/or functional levels. Therefore, morphological and functional levels can be related in the same patient [17–19]. This kind of morpho-functional analysis is of particular interest when dealing with functional disorders of the central nervous system such as migraine. In previous MRI studies we combined water diffusion molecule metrics and analysis of various resting-state brain networks in the same migraine patient and found distinct functional thalamo-cortical connectivity patterns during interictal and ictal periods [20, 21].

In this study, we captured the MRI functional hemodynamics of the cortex at rest to quantify functional connectivity among cerebral independent networks in migraine patients with pure visual auras, and in patients with complex neurological auras. Moreover, the thalamocortical network connectivity was statistically inferred by correlating selected independent networks and thalamic microstructural metrics obtained with diffusion tensor imaging. Given the abovementioned neuroimaging and neurophysiological studies [4–7] and, in particular, our prior interictal VEP studies in similar subgroups of migraine with aura [4], we reasoned that the two subgroups of patients might show both common and distinct neuroimaging abnormalities. We hypothesized that thalamic microstructures would be more impaired in migraine with complex aura than in migraine with pure visual aura, while the functional organization of large-scale neurocognitive networks would be equally affected in both MA subgroups, although thalamocortical network connectivity patterns might be distinct.

## Methods

### Participants

We initially recruited 40 consecutive patients with a diagnosis of migraine with typical aura (ICHD-III code 1.2.1.1) attending our headache clinic. We discarded 5 patients from the analysis because they did not fulfil our strict inclusion criteria, retaining 35 patients (21 women) of Italian ethnicity for the final analysis. The initial patient group was then separated into those who reported pure visual auras (MA, *n* = 20) and those who reported in addition paraesthesia and/or dysphasia (i.e. complex neurological auras; MA+, *n* = 15). We did not include patients with hemiplegic or brainstem aura or persistent aura without infarction. All enrolled patients experienced both migraine attacks with and without aura and their migraine headaches were not side-locked. In order to avoid confounding effects due to pharmacologic treatment, no preventive anti-migraine drugs were allowed during the 3 months preceding the recordings.

We included only patients who were attack-free for at least 3 days prior and 3 days after the day of the MRI session; as mentioned before, this is why we excluded 5 patients from the subsequent analysis. After an ophthalmological evaluation including best-corrected visual acuity, slit-lamp biomicroscopy, intraocular pressure measurement and indirect ophthalmoscopy, only patients without ocular disease were included in the study. We excluded patients with any other type of primary or secondary headache, with a history of other neurological diseases, metabolic disorders, systemic hypertension, and connective or autoimmune diseases.

On the days of screening visit and recording session, we collected the following clinical information from the patients’ headache diary: attack frequency (n/month), duration of migraine history (years), mean severity of migraine attacks (0–10 on visual analogue scale [VAS] score), number of days with acute medication intake (n/month), and number of days elapsed since the last migraine attack (n) (Table 1). We monitored the possible occurrence of a migraine attack within 3 days following the recordings by a telephone call. For comparison, we recruited 19 healthy controls (HC) among healthcare professionals of comparable age and sex distribution as the patients. HC had no personal or family history of migraine or other types of primary headaches, nor any other overt medical condition. Some of the HC used here were already used in previous studies [20, 21]. We managed to scan all female participants at mid-cycle. All MRI sessions were performed in the afternoon (between 4.00 and 7.00 p.m.). Participants were instructed not to drink alcoholic or caffein-containing beverages the day before and on the day of the scanning session, and to refrain from intake of analgesics or other medications.

**Table 1:** Clinical and demographic characteristics of healthy controls (HC), migraine with exclusively visual aura (MA) patients and migraine with complex neurological aura (MA+) patients scanned between attacks. Data are expressed as means ± SD

*Characteristics*	HC (*n* = 19)	MA (*n* = 20)	MA+ (*n* = 15)	Statistics
Female (n)	11	11	10	*χ* = 0.506; *p* = 0.777
Age (years)	28.4 ± 4.1	34.6 ± 10.2	28.8 ± 8.2	*p* = 0.20
Duration of migraine history (years)		15.5 ± 9.7	11.0 ± 6.8	*p* = 0.07
Global attack frequency/month (n)		2.9 ± 2.5	2.5 ± 2.5	*p* = 0.649
Severity of headache attacks (0–10 VAS score)		7.3 ± 1.6	8.0 ± 1.1	*p* = 0.139
Number of acute medication intake/month (n)		2.9 ± 2.5	1.6 ± 1.4	*p* = 0.08
Number of days elapsed since the last attack (n)		15.4 ± 16.4	22.5 ± 12.8	*p* = 0.258
Scintillating scotoma/ Fortification spectra		100 %	100 %	
Sensory symptoms			100 %	
Speech symptoms			26.66 %	

### Ethical approval, and patient consent

 All participants received a complete description of the study and granted written informed consent. The ethical review board of the Faculty of Medicine, University of Rome, Italy, approved the project (RIF.CE 4839).

### Imaging protocols

To obtain functional and structural images, all participants were scanned using a Siemens Magnetom Verio 3T with a 12-channel head coil.

Structural anatomic scans were performed using T1-weighted sagittal magnetization-prepared rapid gradient echo (MP-RAGE) series (repetition time [TR] = 1900 ms, echo time [TE] = 2.93 ms, 176 slices, 0.508 × 0.508 × 1 mm^3^ voxels).

Functional imaging data were collected using a BOLD contrast-sensitive sequence (echo time = 25 ms, flip angle = 90°, resolution = 3.906 × 3.906 × 3 mm); whole-brain echo planar imaging volumes (MRI frames) of 40 contiguous, 3 mm thick axial slices were obtained every three seconds.

Functional BOLD data were obtained in a 7.5-minute run, during which participants were instructed to relax with their eyes closed.

Diffusion tensor imaging (DTI) was acquired by using single shot echo-planar imaging, with an 8–channel head coil (TR 12,200 ms, TE 94 ms, 72 axial slices, 2 mm thickness, isotropic voxels). Images from the same participants and during the same session were computed with diffusion gradients applied along 30 non-collinear directions; effective b values of 0 and 1000 s/mm2 were employed.

### Data processing and analysis

Image data were processed using SPM 12 (http://www.fil.ion.ucl.ac.uk/spm), GIFT v4.0b, FNC (https://trendscenter.org/software) in Mat-Lab environment (www.mathworks.com).

SPM 12 was used to pre-process the data in the following steps.

Single participant EPI images were realigned using a 6-parameter rigid body process, resliced by a cubic spline interpolation.

The structural (T1 – MPRAGE) and functional data were co-registered for each participant dataset. The normalization procedure transformed structural and realigned EPI images into a common stereotactic space based on Talairach and Tournoux [22], resampled by 3 mm on each direction.

Finally, the spatially normalized functional images were smoothed isotropically at 8 mm x 8 mm x 8 mm.

### Group independent component analysis

Grouped spatial independent component analysis (ICA) was performed for all 55 participants using the infomax algorithm [23].

Three separate grouped spatial ICAs were also performed in HCs and MA patients’ subgroups to ensure that the resulting components had similar resting state fluctuations in the three groups as in the resulting components obtained from all 55 participants combined.

Therefore, data were automatically decomposed into 30 components by GIFT software.

A priori probabilistic maps provided by GIFT were used to inspect all 30 components and those of interest whose patterns consisted above all of gray matter rather than non-gray matter were selected [24].

**Fig. 1:**
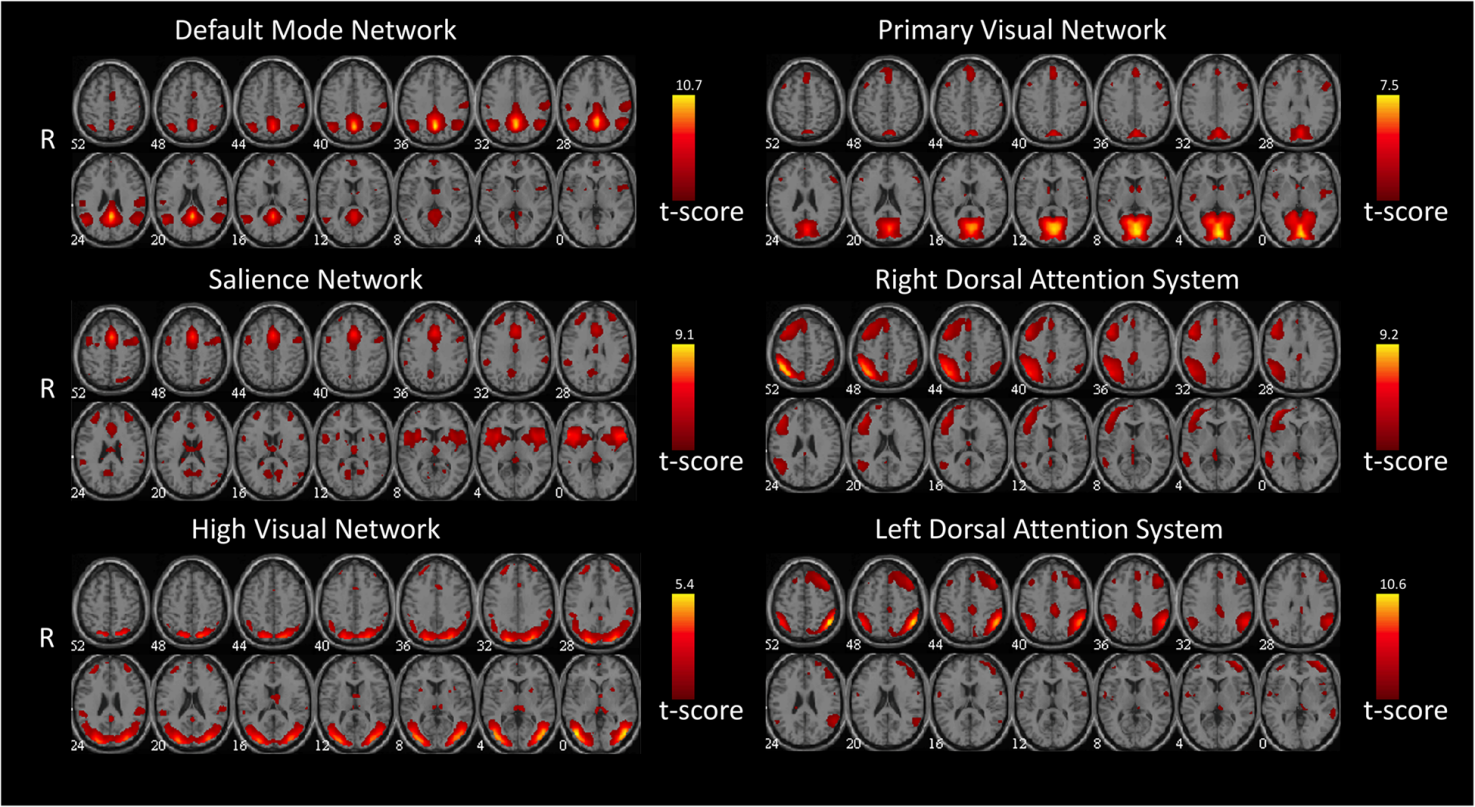
Representation of the 6 selected meaningful independent components. All images have been co-registered into the space of the MNI template. The numbers below each image refer to the z coordinate in Talairach’s.

Following recent guidelines, two experienced neuroradiologists (E.T. & F.C.) blindly reviewed the components and discarded those located in cerebrospinal fluid (CSF) or white matter, or with low correlation to grey matter, since they can be of an artefactual nature (eye movements, head motion, ballistic artefacts) [25]. This process resulted in six meaningful independent components that, corresponded to the following networks [25]: default-mode network (DMN), salience network (SN), high visual, primary visual, and right and left dorsal attention system (DAS), to be processed by means of FNC toolbox (Fig. 1).

With the FNC toolbox in MatLab, only two independent components showed different correlation between groups; they were located in the default mode network (IC15, DMN), and right dorsal attention system (IC24, DAS).

The resulting component time courses were band-pass filtered between the frequencies of 0.017 and 0.067 Hz.

Correlation and lag for this pair of resulting ICs were computed for MA patient subgroups, HCs, and their possible differences, as reported elsewhere [26].

Each IC consists of a temporal waveform and an associated spatial map; the latter is expressed in terms of Z-scores that reflect the degree to which a given voxel time-course correlates with the specific IC temporal waveform, i.e. a way to quantify and measure the strength of the IC [27].

Furthermore, to search for a correlation between regional RS-fMRI network changes, clinical features and DTI metrics, the Z-max scores (voxel-wise analysis) of each IC network were extracted for each participant.

### Diffusion tensor imaging analysis

Image data processing was performed with the FSL 6.0 software package (FMRIB Image Analysis Group, Oxford, England; https://fsl.fmrib.ox.ac.uk/fsl/fslwiki).

Diffusion data were corrected for susceptibility and eddy current distortions, FDT (FMRIB’s Diffusion Toolbox) was employed for local fitting of diffusion tensors. DTI metrics maps were created: FA (fractional anisotropy), MD (mean diffusivity), AD (axial diffusivity), and RD (radial diffusivity).

Two regions of interest (ROI) were defined for each subject, covering the thalamus on the right and left sides of each slice. The medial boundaries on each slice were determined using the CSF as limit, while lateral boundaries were ascertained using FA maps to exclude the internal capsule.

 We calculated mean FA, MD, AD, and RD values in each region for every participant by averaging voxels included in the ROI.

### Statistical analysis

Chi-square test for number of females and t-test with multiple comparison correction (Tukey) for the other demographic and clinical features were used to compare the groups (see Table 1).

### Functional connectivity

We analysed differences in ICs’ correlation between HCs and MA patients, and between HCs and MA + patients using a 2-sample t-test, choosing a p-value of 0.05 false discovery rate corrected (FNC toolbox).

Moreover, connectivity combinations with statistically significant (*p* < 0.05) lag values were also explored using a two-sample t-test of the difference between each averaged contrast: HCs and MA patients, HCs and MA + patients, MA and MA + patients lags.

### Diffusion tensor imaging metrics

FA, MD, AD and RD descriptive statistics of the right and left thalamus were calculated for HCs, MA and MA + patients.

Sample size calculations were based on our previous studies and on a preliminary sample of participants (HC n=10, MA n=9, MA+ n=7). We used the AD and MDvalues for each thalamic ROI to compute the sample size. Comparing HC, MA values and HC, MA+, the minimal required sample size was calculated to be 19participants for HC and MA, and 15 for MA+ (α = 0.05 and β = 0.20). One-way analysis of variance (ANOVA) was performed for each ROI and each DTI metrics mean, in HCs and patients’ subgroups.

We compared the DTI metrics of HCs, MA, and MA + patients in more detail, using a 2-sample t-test corrected for multiple comparisons with Tukey’s method.

We correlated linearly IC 15 and 24 Z-max scores of each participant with the corresponding FA, MD, AD, and RD mean values for each thalamic ROI.

Finally, mean DTI metrics values of each subject were correlated with the corresponding clinical features using Pearson’s test for each ROI.

A p-value of 0.025 was considered significant (0.05/N, where N is the number of ROIs included).

## Results

All subjects completed the recording session. The clinical and demographic data of study participants are shown in Table 1. Analysis of structural brain MRI sequences revealed no white matter lesions.

### Resting state functional connectivity

**Fig. 2:**
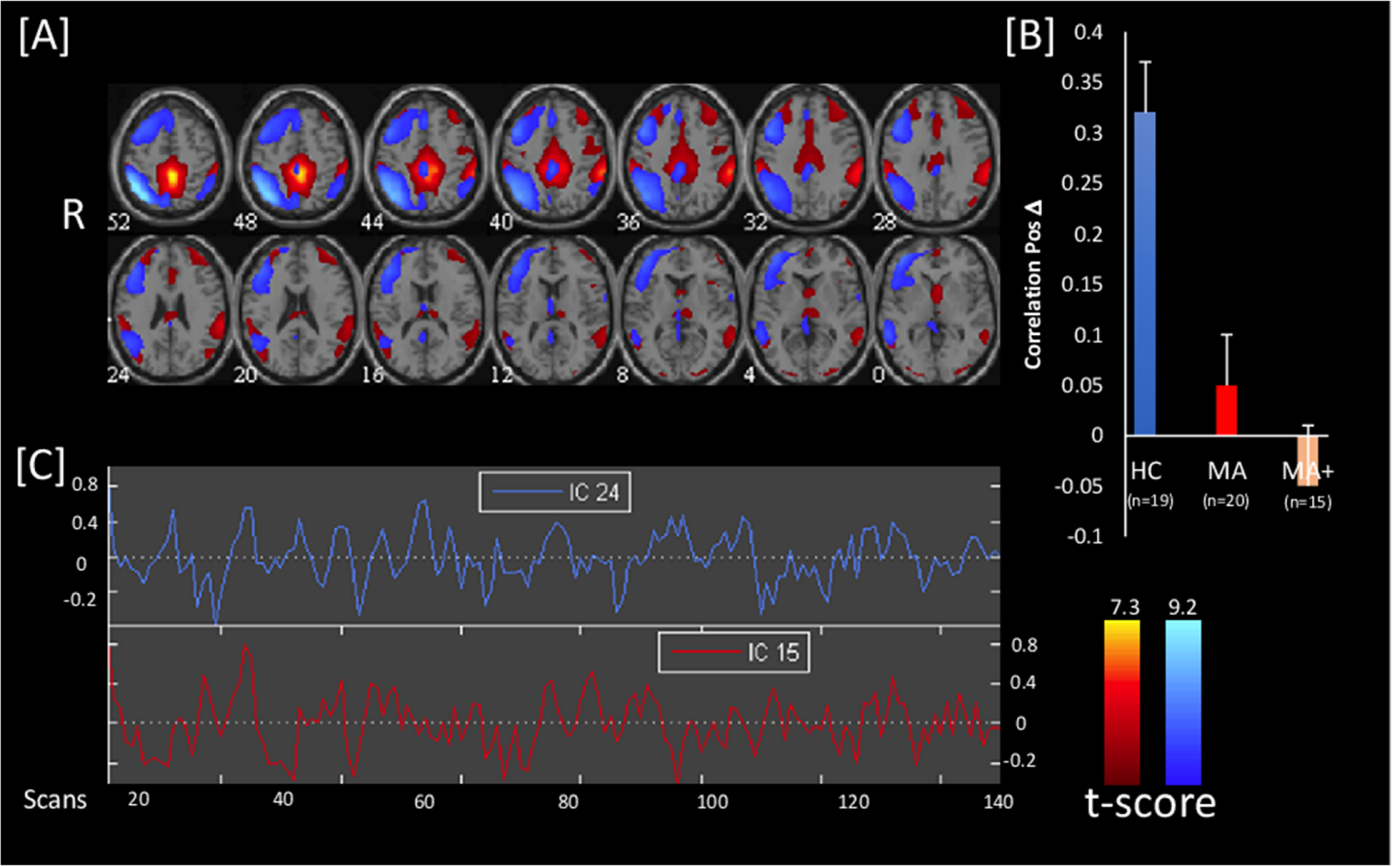
[**A**] Brain distribution of the two Independent Components IC15 (hot metal scale) and IC24 (azure-blue) identified as significant by independent component (IC) analysis and for which functional connectivity was absent in both migraine with pure visual aura (MA) and migraine with complex neurological aura (MA+) patients scanned between attacks and compared to healthy controls (HC). All images have been co-registered into the space of the MNI template. The numbers below each image refer to the z coordinate in Talairach’s atlas. [**B**] the bar graph on the right shows the correlation between the 2 ICs in HC, MA, and MA+, *p* < 0.05 FDR corrected. [**C**] Time course of spontaneous blood oxygen level dependent (BOLD) activity oscillations during resting state, extracted from the two significant ICs.

In the HC group, we found a significant positive correlation between independent components IC15 and IC24 (0.31; *p* < 0.001), encompassing the DMN and right DAS respectively, and a significant lag difference between the two (Fig. 2). This functional connectivity was disrupted in both subgroups of migraine with aura patients (0.042; *p *= 0.372 and − 0.039; *p* = 0.571 in MA and MA+, respectively). The contrasts between HCs and MA patients, and between HCs and MA + patients, were statistically significant (0.265; *p* < 0.001 and 0.345; *p* < 0.001, respectively). However, the contrast between the two subgroups of patients (MA vs. MA+) concerning the independent component pair (IC15-IC24) was not significant (0.08; *p* = 0.286). No significant lag difference was detected for the contrasts listed above. There were no significant correlations between Z-max networks scores and the clinical features of migraine patients.

### Diffusion tensor imaging metrics

None of the diffusivity metrics (FA, MD, RD, and AD) in bilateral thalami of MA patients differed from those of HCs. In MA + patients, on the contrary, MD, AD, and RD values of the right thalamus were significantly lower than those of MA patients (*p* = 0.001, *p* < 0.001, *p* = 0.002 respectively), while MD, AD, and RD values of the left thalamus were significantly lower than those of both HCs (*p* = 0.020, *p* = 0.023, *p* = 0.020 respectively) and MA patients (*p* = 0.006, *p* = 0.006, *p* = 0.007 respectively) (Table 2). There were no significant correlations between DTI metrics and the following clinical features of migraine patients: attack frequency, duration of migraine history, mean severity of migraine attacks, number of days with acute medication intake or number of days elapsed between the recordings and the last migraine attack.

**Table 2:** Diffusion tensor imaging (DTI) metrics of bilateral thalami of healthy controls (HC), migraine with exclusively visual aura (MA) patients, and migraine with complex neurological aura (MA+) patients scanned between attacks. Data are expressed as means ± SD; * MA + vs. HCs *p* < 0.025, ** MA + vs. HCs and vs. MA *p* < 0.025

*DTI metrics*	HC (*n* = 19)	MA (*n* = 20)	MA+ (*n* = 15)
		Right thalamus	
Fractional anisotropy	0.3483 ± 0.02864	0.3372 ± 0.02512	0.3566 ± 0.3286
Mean diffusivity	0.00118 ± 0.00011	0.00123 ± 0.00008	0.00109 ± 0.00008 *
Axial diffusivity	0.00152 ± 0.00009	0.00158 ± 0.00009	0.00145 ± 0.00007 *
Radial diffusivity	0.00100 ± 0.00012	0.00105 ± 0.00008	0.00092 ± 0.00009 *
		Left thalamus	
Fractional anisotropy	0.3379 ± 0.02513	0.3341 ± 0.02881	0.3541 ± 0.03359
Mean diffusivity	0.00122 ± 0.00010	0.00123 ± 0.00010	0.00111 ± 0.00010 **
Axial diffusivity	0.00156 ± 0.00010	0.00157 ± 0.00008	0.00146 ± 0.00008 **
Radial diffusivity	0.00104 ± 0.00011	0.00105 ± 0.00011	0.00093 ± 0.00011 **

### Thalamo-cortical network correlation analysis

**Fig. 3:**
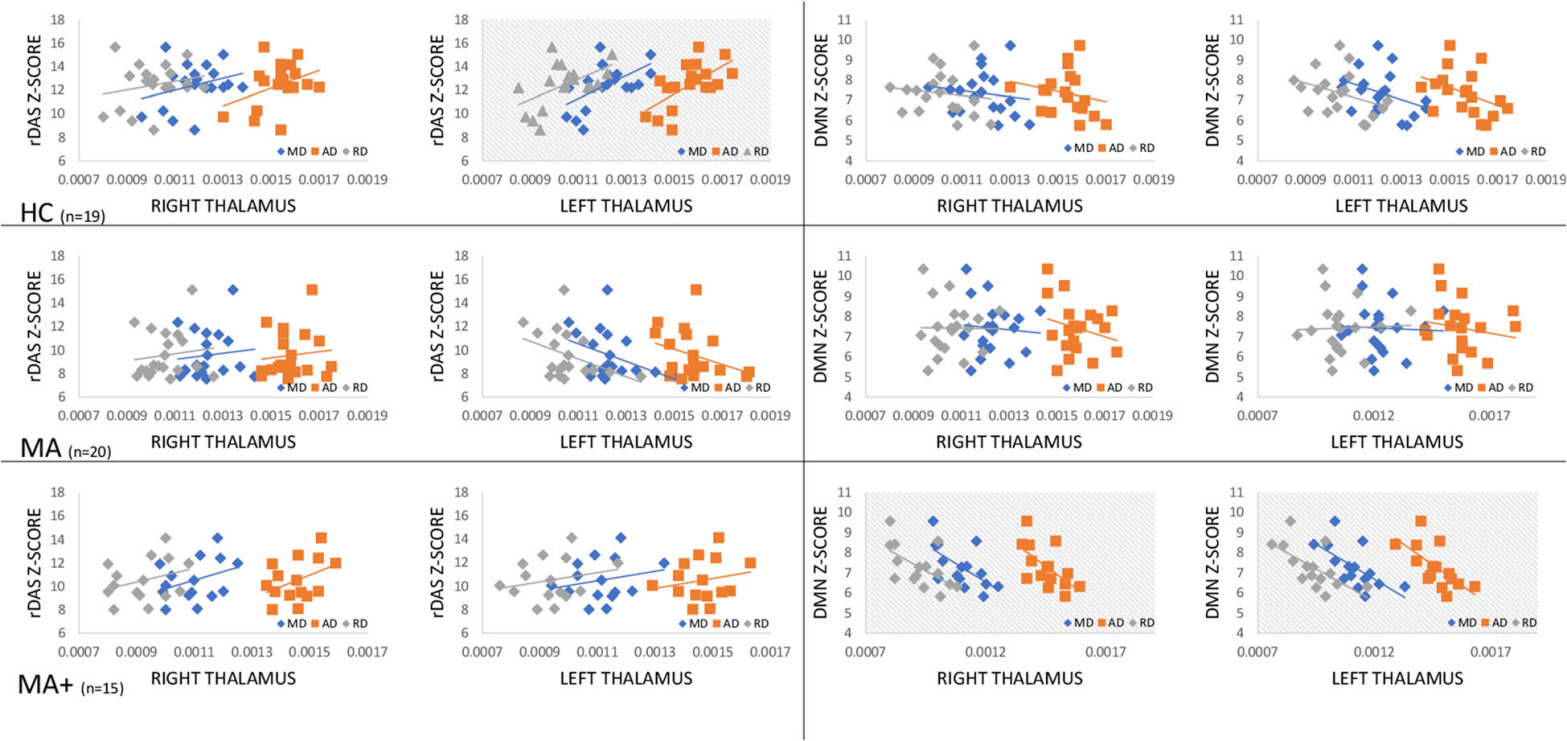
Correlations between Z-scores of IC24 (left panels), encompassing the right dorsal attention system (rDAS), and IC15 (right panels), encompassing the default mode network (DMN), with mean diffusivity (MD), axial diffusivity (AD), and radial diffusivity (RD) values in right and left thalamus in HCs (upper panels), migraine with pure visual aura patients (middle panels) and migraine with complex aura patients (lower panels). The statistically significant correlations are highlighted with a pattern filler.

In HCs, the IC24 Z-score correlated positively with left thalamic MD (F = 8.40, *p* = 0.012, R^2^ = 37.50 % and R^2^adj = 33.04 %; IC 24 = -1.95 + 11,834 MD), AD (F = 10.47, *p *= 0.006, R^2^ = 42.80 % and R^2^adj = 38.71 %; IC 24 = -7.66 + 12,877 AD), and RD (F = 7.43, *p* = 0.016, R^2^ = 34.66 % and R^2^adj = 30.00 %; IC 24 = 0.86 + 11,114 RD) values (Fig. 3). In MA + patients, the IC15 z-score correlated negatively with right thalamic MD (F = 7.09, *p* = 0.021, R^2^ = 37.13 % and R^2^adj = 31.89 %; IC 15 = 15.28–7288 MD), AD (F = 7.45, *p *= 0.018, R^2^ = 38.32 % and R^2^adj = 33.18 %; IC 15 = 19.96–8721 AD), and RD (F = 6.83, *p *= 0.023, R^2^ = 36.26 % and R^2^adj = 13.74 %; IC 15 = 13.48–6724 RD) values, and left thalamic MD (F = 8.17, *p* = 0.014, R^2^ = 40.51 % and R^2^adj = 35.55 %; IC 15 = 14.97–6914 MD), AD (F = 9.06, *p* = 0.011, R^2^ = 43.03 % and R^2^adj = 38.28 %; IC 15 = 19.23–8169 AD), and RD (F = 8.18,* p* = 0.014, R^2^ = 40.53 % and R^2^adj = 22.68 %; IC 15 = 13.37–6512 RD) values (Fig. 3). In MA patients, however, we did not detect any significant correlation.

## Discussion

In the present study we searched for differences in interictal thalamocortical network connectivity between two migraine with aura subgroups, patients with purely visual auras (MA) and patients with complex neurological auras (MA+). The key novel results of this DTI-fMRI study can be summarized as follows: (a) compared to healthy controls (HCs), functional connectivity between the default mode network (DMN-IC15) and the right dorsal attention network (DAS-IC24) is disrupted in both subgroups of patients, (b) metrics of thalamic diffusivity differ significantly between patients with MA + and HCs, but also between the two subgroups of patients, (c) the strength of the DAS (IC24) connectivity correlates positively with certain thalamic diffusivity metrics in HCs, while the strength of DMN (IC15) connectivity correlates negatively with DTI metrics of bilateral thalami in MA + patients.

To the best of our knowledge, this is the first study combining DTI and functional MRI to study thalamocortical network activity in patients with different migraine aura phenotypes. Previous DTI and resting-state fMRI studies were performed in migraine with aura patients without phenotypic distinction.

### Between-network functional connectivity

All previous studies, except one, [28] showed evidence for abnormal cortical functional connectivity [13–15] in migraine with aura patients, both between and during attacks. In a hypothesis-driven resting state fMRI study, Tedeschi et al. [13] isolated the independent component representing the visual network and found a significantly increased activity in the right lingual gyrus (Brodmann’s area 19) of migraine with aura patients, as compared to migraine without aura patients and HCs, but no differences at the macrostructural (grey matter) and microstructural (white matter) level. Comparing migraine patients with and without aura, the connectivity strength of the attention network was found to be slightly higher bilaterally in patients experiencing aura [14]. More recently, patients with complex auras were reported to have a thicker cortex in bilateral visual and somatosensory cortices, and bilateral visual area V5 than patients with simple aura [7]. A major limitation of the two latter studies is lack of a control group, so that it is not clear to which extent presence and complexity of the aura have impacted imaging features of the normal brain [7, 14]. In a recent resting state study using 1.5T fMRI, Veréb et al. [16] detected a non-significant trend of weaker causal interaction from the DMN to the DAS in migraine with aura in comparison to HCs.

Along the same line, we show here that migraine with aura patients have disrupted functional connectivity between the DMN and the DAS compared to HCs, irrespective of reporting pure visual aura or complex aura with additional paraesthesia and/or dysphasia.

While the DMN is devoted to more internally focused tasks [29], the DAS is more externally focused, being responsible for top-down cognitive selection of relevant sensory information, multimodal stimulus processing – with a predilection for the visual input – and preparation of responses or action selection [30, 31]. It is well known that DMN and DAS are distinct and functionally competitive networks.[30] Resting-state data systematically indicate that spontaneous DMN activity is physiologically deactivated by attention-demanding tasks and anticorrelated to that of DAS [32]. Therefore, an abnormal connectivity between DAS and DMN could contribute to brain dysfunction [33, 34].

It is of interest that an abnormal connectivity between the self-orientation monitoring network and the externally-oriented multimodal sensory information processing networks seems to be a general characteristic of patients with migraine. In our previous fMRI studies in migraine without aura patients, we found a large-scale reorganization of functional connectivity between the DMN and the visuo-spatial system interictally [20], between the executive control (ECN) and the dorso-ventral attention networks during an attack [21], and between the DMN, DAS, and ECN in patients with chronic migraine [35, 36] compared to HCs. Moreover, we detected specific correlation patterns between metrics of thalamic microstructure and strength of cortical networks [20, 21].

### Thalamic microstructure

In a whole brain analysis, migraine with visual aura patients had lower FA values in the lateral geniculate nucleus [37]. In another study, the same group reported a significantly shorter T1 relaxation time – a measure of iron deposition and cellularity – but normal FA values, in the thalamus of all but two migraine with visual aura patients compared with HCs [10]. In agreement with the first study, we found no statistical difference in diffusivity measures between our subgroups of MA patients and HCs. By contrast, MA + patients had significantly lower MD, AD, and RD values in the left thalamus as compared to HCs or MA, and in the right thalamus as compared to MA. The MD metric comprises RD and AD, and quantifies the overall magnitude of water diffusion by indicating both cellular swelling and cellular density [38]. In particular, AD and RD are considered to be *in vivo* surrogate markers of myelin and axonal damage, respectively.

The MD, AD, and RD decrease found in MA + patients, may reflect a slight decrease in cellularity (neuronal and glial) and/or a gain in directional organization of highly anisotropic myelinated fibres interconnecting individual thalamic nuclei [39].

### Thalamo-cortical network connectivity

Group-specific features also resulted from the correlation analysis between the microstructural and the functional variables. In HCs, the strength of the DAS connectivity was positively related to the MD, AD, and RD diffusivity metrics of the left thalamus. Such correlation was absent in both MA and MA + subgroups. MA + was the only subgroup, where the DMN Z-score was anti-correlated with the MD, AD, and RD DTI metrics of the bilateral thalamus.

Previous studies have found that the function of the thalamus is coordinated by multiple regions of the brain [40]. From resting-state fMRI studies in healthy controls, the thalamus is known to be broadly connected with the cortex, not only to individual cortical lobes, but also to a set of spatially distinct cortical regions supporting similar functions, i.e. organized in networks. Among the thalamic nuclei, the pulvinar and the lateral posterior nucleus are associated with both the DAS and the visual networks, whereas the DMN is associated with the anterior nucleus, the medial dorsal nucleus, and the pulvinar [41].

With this pattern of thalamocortical connectivity in mind, we may hypothesize that the MRI pattern of normal thalamic microstructure and disrupted thalamocortical relationship detected in our MA patients could be due either to a pure cortical alteration, or to an alteration of thalamocortical fibre bundles. Previous studies in patients with exclusively visual aura showed widespread disruption of white matter fibre bundle architecture [8, 9, 11], which could contribute to the between-network disconnection found in our patients and in those from other research groups [12, 16].

In MA + patients the intrinsic microstructural abnormalities of the thalami and the distinct anti-correlation with the DMN that in turn is disconnected from the DAS, suggest a more widespread involvement of both the thalamic nuclei and the cerebral cortex associated with a phenotypically more complex form of migraine aura. This hypothesis is supported by imaging studies showing that, as compared to exclusively visual auras, complex auras are accompanied by more extended vascular changes overspanning major adjacent cerebral vascular territories [42–44].

### Relevance for migraine with aura pathophysiology

In MR spectroscopy studies, resting and stimulation-evoked metabolic abnormalities of the visual cortex differ between patients with different aura phenotypes, being most pronounced in those with more complex auras [6, 45]. Evoked magnetic and electric responses of the visual cortex are greater in patients with complex or prolonged auras than in those with pure visual auras, although habituation of the visual responses during sustained stimulation is deficient in both patient groups [4, 46]. Whether and how these specific and common interictal electrophysiological abnormalities are related to the specific thalamic/thalamo-cortical and common between-network aberrant connectivity we found in the present study remains to be determined.

Moreover, whether the observed abnormal thalamo-cortical network connectivity patterns between attacks are related to the ictal neurovascular phenomenon of cortical spreading depression (CSD), the likely culprit of the migraine aura, remains speculative. In rodents, CSD can modify for long durations the firing rate of thalamic neurons controlling the flow of sensory information to the cortex [47], independently from concurrent peripheral trigeminal inputs [48]. In knock-in mice expressing the S218L mutation in the CACNA1A gene that causes familial hemiplegic migraine type 1, clinical phenotype, susceptibility to CSD and its subcortical spread down to the thalamus are more pronounced than in mice carrying the R192Q mutation that induces less severe clinical symptoms [49]. Repeated, extensive and long-lasting activation of the cortico-thalamic pathway by CSD could induce or worsen interictal impairment of thalamic activity, which may reflect in plastic changes at the microstructural level, as seen with DTI in migraine patients with complex auras. One may argue, however, that in this scenario a positive correlation would be expected between the interictal thalamocortical functional and structural changes and attack frequency, which was not the case when number of all migraine attacks were considered, unless these changes tend to normalize as time since the last attack elapses.

## Limitations

Our study has several limitations. First, due to the short clinical follow-up prior to the recording session, we were not able to collect reliable information separately about frequency and duration of attacks with aura, both of which might be more relevant clinical correlates for thalamo-cortical network changes than combined frequency and duration of both with and without aura attacks. Second, this a cross-sectional study on a relatively small cohort of subjects and with retrospective collection of clinical data. A larger cohort of migraine patients with various clinical phenotypes and a longitudinal, prospective follow-up would allow for a more reliable comparison of MRI and clinical data, like for instance the frequency of the auras, and for assessing dynamic changes at different time points of the migraine cycle. Fourth, we did not collect simultaneous EEG activity, which would have allowed us to exclude individual variations in the level of alertness and the occurrence of microsleeps that can be possible sources of variability in thalamocortical functional connectivity. Furthermore, healthcare professionals, who were a large part of our healthy control group, might have of different socioeconomic background than most patients, and may have had more years of education, either of which may be associated with different functional connectivity.

## Conclusions

In summary, this study shows that clinical heterogeneity of migraine with aura MRI profiles is associated with common and specific morpho-functional features of the nodes of the thalamo-cortical network. We found disrupted functional connectivity between DMN and right DAS equally in both MA and MA + patients compared to HCs. MA + subgroup of patients showed lower microstructural metrics than those of both HCs and MA, and peculiar correlation with the strength of DMN. Despite the microstructural metrics of MA patients did not differ from those of HCs, they did not show the same correlations with the strength of DAS than HCs.

Finally, whether these distinct results of the two subgroups of patients are primary related to the CSD features or to a different genetic load that may act on both CSD and MRI profile remains to be determined.

## Data Availability

The informed consent form signed by all participants in this study did not include a provision stating that individual raw data can be made publicly accessible. Therefore, in agreement with the Italian data protection law, individual de-identified participant raw data cannot be publicly shared. Researchers meeting the criteria for access to confidential data may access the data upon request.

## Abbreviations

AD: axial diffusion
CC: corpus callosum
CM: chronic migraine
DTI: diffusion tensor imaging
FA: fractional anisotropy
HC: healthy control
IC: internal capsule
ICHD: International Classification of Headache Disorders
LF: longitudinal fasciculus
MD: mean diffusion
MO: episodic migraine without aura
PCR: posterior corona radiata
RD: radial diffusion
SCR: superior corona radiata
TBSS: tract-based spatial statistics
VAS: visual analogue scale
WM: white matter
